# Long-Term Correction of Nasolabial Folds Using Poly-L-Lactic Acid Microspheres: A Multicenter, Double-Blinded, Randomized Trial

**DOI:** 10.1093/asjof/ojag001

**Published:** 2026-01-13

**Authors:** Zhaojian Wang, Wenyun Ting, Hongyi Zhao, Lei Huang, Jianming Ma, Hongwei Liu, Yanjun Liang, Maoguo Shu, Lin Jin, Nanze Yu, Xiao Long

## Abstract

**Background:**

Correction of moderate-to-severe nasolabial folds (NLFs) is a key concern in aesthetic medicine. Poly-L-lactic acid (PLLA) microspheres, with biostimulatory properties, potentially offer longer-lasting results compared with hyaluronic acid (HA), the current standard of care.

**Objectives:**

The aim of this study was to evaluate the efficacy and safety of PLLA microspheres vs HA for the correction of moderate-to-severe NLFs.

**Methods:**

In this multicenter, randomized, double-blind, parallel-controlled trial, 252 participants were enrolled across 8 centers in China and assigned to receive either PLLA or HA filler. The primary endpoint was the effective correction ratio of the Wrinkle Severity Rating Scale (WSRS) at 48 weeks, assessed by blinded independent reviewers. Secondary endpoints included WSRS change, Global Aesthetic Improvement Scale (GAIS) scores (investigator- and participant-assessed), treatment frequency, and safety outcomes.

**Results:**

At 48 weeks, PLLA demonstrated a significantly higher effective correction ratio than HA (92.4% vs 59.3%; difference, 33.1% [95% CI, 23.1-43.2]; *P* < .0001). HA outperformed PLLA at Week 4, reflecting its immediate effect, whereas PLLA showed superior outcomes from Week 36 onward with sustained improvement confirmed by WSRS and GAIS. PLLA required more frequent early treatments, whereas HA generally achieved correction with fewer sessions. Adverse events were mild and transient, with no serious complications in either group.

**Conclusions:**

Under current protocols, HA offers rapid improvement, whereas PLLA requires multiple early sessions but yields more durable correction up to 48 weeks with similar safety. Filler selection should be individualized according to patient priorities.

**Level of Evidence: 2 (Therapeutic):**

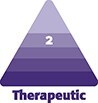

Minimally invasive aesthetic procedures have grown rapidly worldwide, with injectable fillers representing a major segment of the aesthetic market.^1^ These treatments are valued for their efficacy, short recovery time, and versatility in addressing age-related facial changes such as volume loss, rhytides, and contour deficiencies. Among these, dermal fillers have become a mainstay in nonsurgical facial rejuvenation, offering immediate or delayed volumization depending on the material properties.

Injectable poly-L-lactic acid (PLLA) is a synthetic, biodegradable polymer that achieves soft tissue augmentation through a mechanism distinct from traditional fillers. Unlike hyaluronic acid (HA) fillers, which restore volume immediately through hydrophilic effects, PLLA acts through foreign-body responses involving macrophage recruitment and fibroblast activation.^2,3^ These cellular events are hypothesized to result in encapsulation of PLLA particles with newly deposited collagen and gradual tissue remodeling, producing progressive volumetric improvement.^4^ Reported effects have been observed to last up to 2 years.^5,6^

Since its first approval in Europe in 1999 and subsequent US FDA approval in 2004 for HIV-associated lipoatrophy, PLLA has been increasingly used in aesthetic practice.^6,7^ Emerging evidence suggests that, in addition to volumization, PLLA may improve skin elasticity and dermal quality over time, with a generally favorable safety profile.^8,9^ Nonetheless, existing comparative studies between PLLA and HA have been limited by small sample sizes, short follow-up (≤24 weeks), and heterogeneous designs, leaving questions regarding their relative temporal and long-term efficacy unresolved.

Against this background, rigorous evaluation of long-acting fillers is particularly necessary. This multicenter, randomized, double-blind, parallel-controlled clinical trial aims to directly compare PLLA and HA for the correction of moderate-to-severe nasolabial folds (NLFs). By assessing both short-term and long-term outcomes over 48 weeks, the trial seeks to clarify the temporal efficacy profiles and safety of these 2 agents, thereby providing evidence to guide clinical decision making in filler selection.

## METHODS

### Study Design

This multicenter, randomized, double-blinded, parallel-controlled clinical trial was conducted across 8 centers in China between July 2022 and April 2024. The study was prospectively registered with the Jiangsu Medical Product Administration (No. 苏械临备20220140), and the protocol was reviewed and approved by the regulatory authority. The aim was to evaluate the efficacy and safety of injectable PLLA microspheres (Xihong Biopharma, Jiangsu, China) for the correction of moderate-to-severe NLFs. Modified sodium hyaluronate gel (Restylane, Q-Med AB, Uppsala, Sweden) served as the control treatment. The study adheres to the guidelines of the National Medical Products Administration and the ethical standards of the Declaration of Helsinki. The study has been approved by the Ethical Review Committee of Peking Union Medical College Hospital (no. KS2022094), Beijing Hospital (2022BJYYEC-073-02), Xianyang Hospital of Yan’an University (X-XJTU1AF2022LSY-74), Linfen Central Hospital (QX2022-K202202-02), The First Affiliated Hospital of Jinan University (YDXYEC-YWPJ-2022(18)), Taiyuan City Central Hospital (LFCH-IRB-PJ-QX-2022(5)), and The First Affiliated Hospital of Xi'an Jiaotong University (2022-LSX005-1). Written informed consent was obtained from all participants before participation.

### Participants

Participants were recruited through institutional poster advertisements and online postings and volunteered to participate. No costs were incurred by participants. The study was financially supported by China Medical System Holdings Limited, which provided funding only. The sponsor had no involvement in study design, study conduct, data collection, data analysis/interpretation, manuscript preparation, or journal selection. Eligible participants were Chinese adults aged ≥18 years with moderate-to-severe NLFs (Wrinkle Severity Rating Scale [WSRS] 3-4) who refrained from aesthetic treatments during the study, adhered to protocols, and provided informed consent. Exclusions included pregnancy/breastfeeding, contraception refusal in women of childbearing potential, visual impairments affecting assessments, clinically significant laboratory anomalies, positive HBsAg/anti-HCV/anti-HIV/anti-TP, treatment-area skin disorders, previous permanent NLF fillers, facial/aesthetic surgeries or semi-permanent fillers (eg, HA) within 1 year, wrinkle treatments (eg, lasers, botulinum toxin) within 6 months, anticoagulant/antiplatelet use ≤14 days pretreatment, lidocaine/filler allergies, severe systemic/autoimmune diseases, keloid history, recent trial participation (≤30 days), or investigator-determined unsuitability (Appendix).

### Sample Size Calculation, Randomization, and Blinding

Sample size estimation was based on a superiority margin of 20% between the groups. Assuming an 80% power and a 1-sided significance level of 0.015, 124 participants per group were required (Appendix). Randomization was conducted using an electronic centralized randomization system for clinical trials. Each center applied a block randomization method to assign random numbers, ensuring balanced allocation. Eligible participants were randomly assigned in a 1:1 ratio to either the PLLA or HA group. Both participants and independent evaluators were blinded to the treatment allocation. Products were prepared and administered by treating physicians who were not involved in subsequent outcome assessments. In addition, syringes were masked to conceal product identity before injection.

### Intervention Procedures

Local anesthesia with topical lidocaine cream was applied before all injections. In the PLLA group, 5 mL of sterile saline was mixed with 150 mg PLLA powder and allowed to stand for 20 min before injection. The reconstituted filler was injected into the deep dermal or subdermal layer using a retrograde injection technique with a 26G needle. Aspiration was performed before each injection to ensure the needle was not intravascular. The product was delivered slowly while the needle was withdrawn, and the injection was terminated before complete withdrawal of the needle from the skin. Injections were placed at ∼1.0 to 1.5 cm intervals, with each aliquot limited from 0.1 to 0.2 mL. Injection volumes were determined by treating physicians to achieve optimal correction, with a maximum of 2.5 mL per side. In the HA group, sodium hyaluronate gel was administered using identical procedures with a prefilled 29G syringe. The maximum injection volume was 1.5 mL per side at the initial treatment and 0.5 mL at complementary sessions. Standardized 2D photographs were taken at baseline and each follow-up visit to ensure consistency in outcome assessments. Optimal correction was defined as a WSRS score of 1 on both NLFs or an improvement of at least 2 grades from baseline, in which case no additional treatment was required. To achieve optimal correction, participants were eligible for complementary sessions within 12 weeks of the initial treatment, scheduled every 3 to 5 weeks at the investigators’ discretion, with a maximum of 3 sessions permitted. The study follow-up period (up to 48 weeks) was defined relative to the last treatment session, ensuring that all efficacy and safety assessments reflected the stable outcomes of the completed treatment course rather than transient effects of recent injections.

### Efficacy Assessment

The primary outcome was the effective correction ratio of NLFs at 48 weeks posttreatment, defined as the participant proportion with a reduction of at least 1 grade on the WSRS as assessed by blinded evaluators. The WSRS is a validated 5-point photographic scale specifically developed and validated for NLFs (Supplemental Table 1).^10,11^ Secondary outcomes included WSRS score changes provided by blinded evaluators and Global Aesthetic Improvement Scale (GAIS) ratings provided by both participants and investigators at 4, 12, 24, 36, and 48 weeks after the last treatment.

### Safety Assessment

Adverse events (AEs) were monitored throughout the study, with a focus on treatment-emergent AEs, such as swelling, erythema, pain, and nodules. Severity and resolution of AEs were recorded, and all events were classified using the Medical Dictionary for Regulatory Activities (MedDRA). Serious complications, such as intravascular injection, were specifically monitored.

### Population Sets

Three analysis populations were predefined for this study. The full analysis set (FAS) included all participants who met eligibility criteria, provided written informed consent, received at least 1 injection, and underwent at least 1 postbaseline efficacy assessment, following the intention-to-treat principle. Missing primary endpoint data were imputed using the worst observation carried forward method. The per-protocol set (PPS) included participants who adhered fully to the study protocol, had complete baseline data for the primary endpoint, demonstrated good compliance, and completed the study according to protocol specifications. Primary efficacy analyses were conducted based on FAS in this study. Supportive analyses based on PPS were used to demonstrate consistency. Demographic and baseline characteristics were summarized based on the FAS.

### Statistical Analysis

All analyses were performed using SAS version 9.4 (SAS Institute Inc., Cary, NC). The absolute difference in effective correction ratios between groups was estimated with the Newcombe–Wilson method and corresponding 95% CIs. Superiority for the primary endpoint was assessed using a Cochran–Mantel–Haenszel χ^2^ test adjusted for center effects, with superiority defined as a lower 95% CI bound above 0. Continuous outcomes (eg, WSRS, GAIS) were analyzed as mean ± standard deviation and compared using *t*-tests or Wilcoxon rank-sum tests, as appropriate. Ordinal variables were analyzed with nonparametric methods. Standardized effect sizes were calculated to quantify between-group differences in change scores. All comparisons employed 2-sided tests; a *P-*value of <.05 was considered statistically significant. Safety analyses included all participants who received at least 1 injection, with AEs summarized by frequency, severity, and causality, and compared using χ^2^ or Fisher's exact tests.

## RESULTS

### Patient Demographics

**Figure 1.**
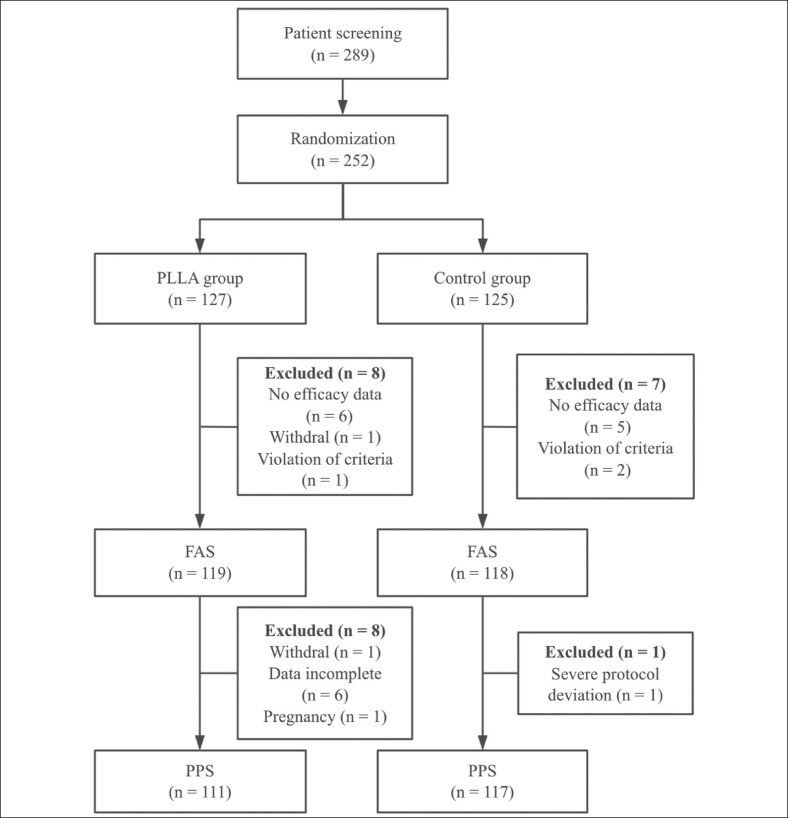
The workflow diagram of the study.

**Figure 2.**
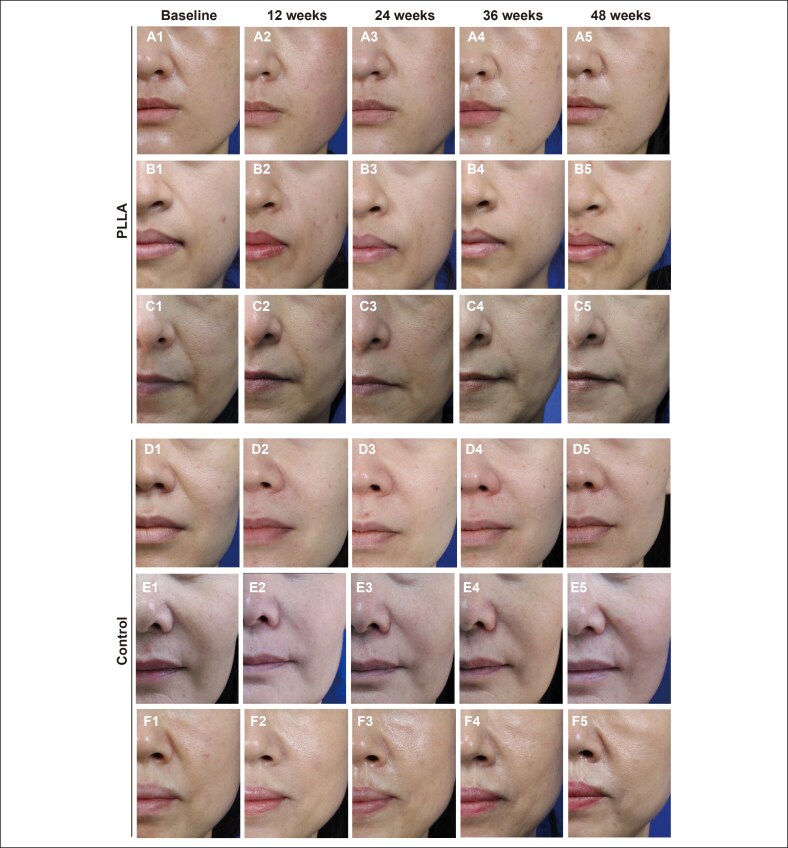
Representative cases receiving poly-L-lactic acid (PLLA) and hyaluronic acid (HA) injection. The nasolabial fold of (A1-A5) a 28-year-old female, (B1-B5) a 35-year-old female, (C1-C5) a 54-year-old male in the PLLA group, (D1-D5) a 40-year-old female, (E1-E5) a 48-year-old female, and (F1-F5) a 53-year-old female in the HA group at baseline, 12 weeks, 24 weeks, 36 weeks, and 48 weeks after the last treatment session, respectively.

A total of 289 volunteers were screened, of whom 252 were enrolled and randomized: 127 participants to the PLLA group and 125 to the HA group (Figure 1). Ultimately, 119 participants (93.7%) in the PLLA group and 118 (92.9%) in the HA group completed the 48-week follow-up (Figure 1). The PLLA group included 115 female and 4 male participants, whereas the HA group included 112 female and 6 male participants. The mean age was 41.8 ± 9.4 years (range, 25.0-64.0) in the PLLA group and 43.3 ± 9.7 years (range, 21.0-65.0 years) in the HA group. Baseline WSRS scores were comparable between groups (Table 1, Figure 2), indicating no significant imbalance in wrinkle severity at study entry. The frequency of treatment sessions differed significantly between groups (*P* < .0001, Supplemental Tables 2, 3). In the HA group, most participants (79.66%) achieved optimal correction with 1 or 2 sessions, whereas in the PLLA group, the majority (74.79%) required 3 or 4 sessions. Complementary sessions were not performed in participants who had already achieved a WSRS score of 1 on both NLFs or demonstrated ≥2-grade improvement from baseline.

**Table 1.** Participant Demographics

Variable	PLLA group	HA group	Overall	*P*-value
	119	118	237	
Gender				.5093
Male, *n* (%)	4 (3.36)	6 (5.08)	10 (4.22)	
Female, *n* (%)	115 (96.64)	112 (94.92)	227 (95.78)	
Age (years old)				.1943
Mean (SD)	41.75 (9.44)	43.31 (9.69)	42.53 (9.57)	
Median (Q1, Q3)	40.00 (35.00, 50.00)	41.50 (36.00, 50.00)	41.00 (35.00, 50.00)	
Min, max	25.00, 64.00	21.00, 65.00	21.00, 65.00	
Height (cm)				.2113
Mean (SD)	162.64 (6.10)	161.89 (5.93)	162.27 (6.01)	
Median (Q1, Q3)	162.50 (159.00, 166.00)	161.50 (158.00, 165.00)	162.00 (158.00, 166.00)	
Min, max	147.00, 186.00	149.50, 183.50	147.00, 186.00	
Weight (kg)				.8675
Mean (SD)	58.20 (8.22)	58.84 (9.46)	58.52 (8.85)	
Median (Q1, Q3)	57.00 (52.90, 63.00)	57.60 (52.00, 64.70)	57.50 (52.50, 64.00)	
Min, max	41.00, 85.30	44.00, 93.00	41.00, 93.00	
WSRS				.3927
Grade 3, *n* (%)	57 (47.90)	50 (42.37)	107 (45.15)	
Grade 4, *n* (%)	62 (52.10)	68 (57.63)	130 (54.85)	

HA, hyaluronic acid; PLLA, poly-L-lactic acid; SD, standard deviation; WSRS, Wrinkle Severity Rating Scale.

### Primary Efficacy Outcome

**Figure 3.**
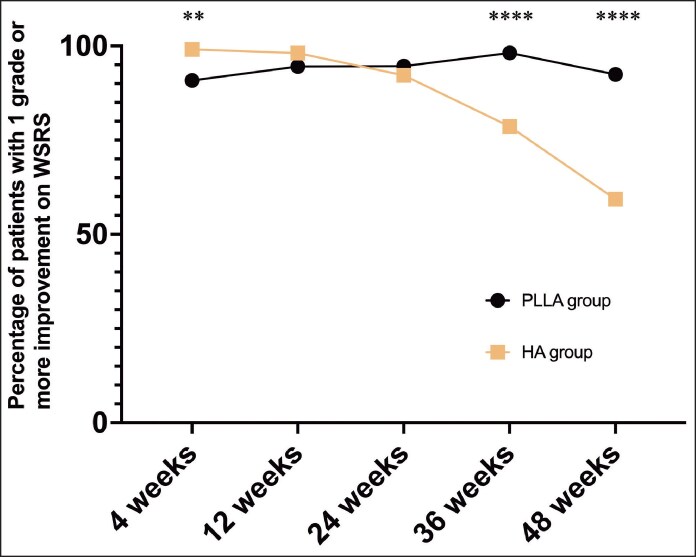
The Wrinkle Severity Rating Scale (WSRS) effective percentage for nasolabial fold wrinkles of poly-L-lactic acid (PLLA) and hyaluronic acid (HA) groups. **P < .01, ****P < .0001.

In the FAS, the effective correction ratio of WSRS at Week 48, as assessed by blinded independent reviewers, was 92.44% in the PLLA group and 59.32% in the HA group, yielding a between-group difference of 33.11% (95% CI, 23.06%-43.17%; *P* < .0001; Table 2, Figure 3). In the PPS, the corresponding ratios were 95.50% and 59.83%, respectively, with a difference of 35.67% (95% CI, 25.98%-45.35%; *P* < .0001; Supplemental Table 4). In both FAS and PPS analyses, the lower bounds of the 95% CIs exceeded the predefined superiority margin of 0%, confirming that PLLA achieved significantly superior efficacy over HA at 48 weeks.

**Table 2.** Comparison of WSRS Effective Ratio Between PLLA and HA Groups

Group	No. of patients with ≥ 1 grade improvement	Effective ratio (%)	Difference (95% CI)	*P*-value
Week 4				
PLLA (*n* = 109)	99	90.83	−8.26% (−15.19%, −2.45%)	.0054
HA (*n* = 109)	108	99.08		
Week 12				
PLLA (*n* = 110)	104	94.55	−3.57% (−9.66%, 2.00%)	.2805
HA (*n* = 106)	104	98.11		
Week 24				
PLLA (*n* = 112)	106	94.64	1.72% (−4.58%, 8.03%)	.5928
HA (*n* = 113)	105	92.21		
Week 36				
PLLA (*n* = 108)	106	98.15	19.52% (11.56%, 27.90%)	<.0001
HA (*n* = 117)	92	78.63		
Week 48				
PLLA (*n* = 119)	110	92.44	33.11% (23.06%, 43.17%)	<.0001
HA (*n* = 118)	70	59.32		

WSRS scores were assessed by blinded independent evaluators. HA, hyaluronic acid; PLLA, poly-L-lactic acid; WSRS, Wrinkle Severity Rating Scale.

### Secondary Efficacy Outcome

#### Effective Correction Ratios

Effective correction ratios based on WSRS at earlier time points are summarized in Table 2 and Figure 3. At Week 4, the HA group achieved a significantly higher effective correction ratio compared with the PLLA group (99.08% vs 90.83%; difference, −8.26% [95% CI, −15.19% to −2.45%]; *P* = .0054). At Week 12, both groups demonstrated high response ratios with no significant difference (98.11% vs 94.55%; *P* = .2805). By Week 24, correction remained comparable (92.21% vs 94.64%; *P* = .5928). At Week 36, however, PLLA demonstrated a markedly higher effective correction ratio (98.15% vs 78.63%; difference, 19.52% [95% CI, 11.56%-27.90%]; *P* < .0001), reflecting the onset of PLLA's longer-term advantage. These trends were similar in the PPS (Supplemental Table 4).

#### WSRS Change From Baseline

Table 3 and Supplemental Figure 1 summarize mean WSRS score changes. At Week 4, HA showed greater improvement than PLLA (−1.75 ± 0.60 vs −1.30 ± 0.65; *P* < .0001; standardized effect size, 0.724 [95% CI, 0.448-0.999]). Differences were no longer significant at Week 12 (*P* = .078) or Week 24 (*P* = .292). By Week 36, PLLA provided superior sustained improvement (−1.31 ± 0.54 vs −1.10 ± 0.77; *P* = .019), with the difference more pronounced at Week 48 (−1.28 ± 0.64 vs −0.83 ± 0.84; *P* < .0001; standardized effect size, −0.599 [95% CI, −0.856 to −0.343]). Similar trends were observed in the PPS (Supplemental Table 5).

**Table 3.** Comparison of WSRS Improvement From Baseline Between PLLA and HA Groups

WSRS	PLLA		HA		*P-*value	Standardized effect size
	*n*	Value of change, mean(SD)	*n*	Value of change, mean(SD)		
Week 4	109	−1.30 (0.65)	109	−1.75 (0.60)	<.0001	0.724 (0.448, 0.999)
Week 12	110	−1.39 (0.65)	106	−1.56 (0.65)	.078	0.255 (−0.014, 0.524)
Week 24	112	−1.29 (0.58)	113	−1.37 (0.64)	.292	0.126 (−0.137, 0.389)
Week 36	108	−1.31 (0.54)	117	−1.10 (0.77)	.019	−0.319 (−0.580, −0.058)
Week 48	119	−1.28 (0.64)	118	−0.83 (0.84)	<.0001	−0.599 (−0.856, −0.343)

WSRS scores were assessed by blinded independent evaluators. HA, hyaluronic acid; PLLA, poly-L-lactic acid; SD, standard deviation; WSRS, Wrinkle Severity Rating Scale.

#### Investigator-Assessed GAIS

**Figure 4.**
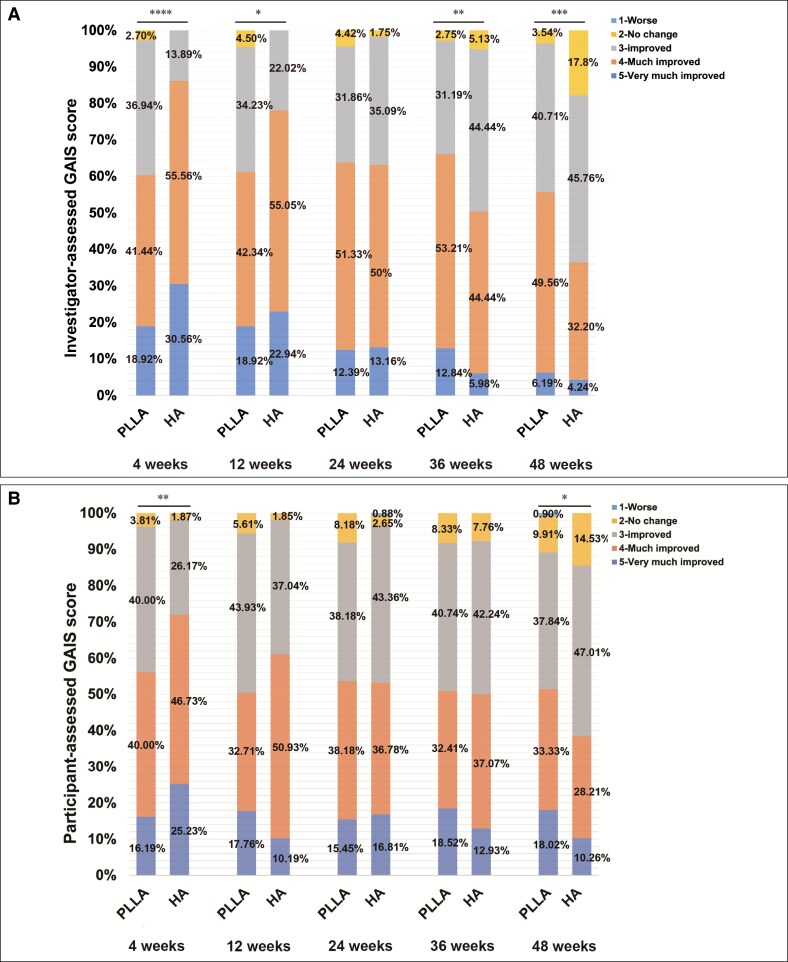
Global Aesthetic Improvement Scale scores of poly-L-lactic acid (PLLA) and hyaluronic acid (HA) groups assessed by investigators (A) and participants (B). *P < .05, **P < .01, ***P < .001, ****P < .0001.

GAIS scores assessed by investigators are presented in Table 4 and Figure 4. At early follow-ups, HA was superior at Week 4 (2.23 ± 0.79 vs 1.83 ± 0.65; *P* < .0001) and Week 12 (2.24 ± 0.81 vs 1.99 ± 0.67; *P* = .018). No difference was observed at Week 24 (*P* = .888). In contrast, PLLA showed greater improvement at Week 36 (2.49 ± 0.69 vs 2.24 ± 0.71; *P* = .008) and Week 48 (2.77 ± 0.79 vs 2.42 ± 0.66; *P* = .0004), consistent with its durable corrective effect.

**Table 4.** Comparison of GAIS Between PLLA and HA Groups

GAIS	PLLA		HA		*P-*value	Standardized effect size
	*n*	Score, mean (SD)	*n*	Score, mean (SD)		
Investigator-assessed						
Week 4	111	2.23 (0.79)	108	1.83 (0.65)	<.0001	0.556 (0.285, 0.827)
Week 12	111	2.24 (0.81)	109	1.99 (0.67)	.018	0.339 (0.073, 0.604)
Week 24	113	2.28 (0.74)	114	2.25 (0.70)	.888	0.040 (−0.221, 0.301)
Week 36	109	2.24 (0.71)	117	2.49 (0.69)	.008	−0.356 (−0.620, −0.092)
Week 48	113	2.42 (0.66)	118	2.77 (0.79)	.0004	−0.486 (−0.749, −0.223)
Participant-reported						
Week 4	111	2.34 (0.79)	108	2.04 (0.77)	.004	0.390 (0.122, 0.659)
Week 12	111	2.38 (0.85)	109	2.30 (0.67)	.366	0.098 (−0.167, 0.364)
Week 24	113	2.41 (0.85)	114	2.35 (0.82)	.684	0.067 (−0.194, 0.329)
Week 36	109	2.39 (0.88)	117	2.45 (0.81)	.663	−0.069 (−0.331, 0.193)
Week 48	113	2.42 (0.92)	118	2.66 (0.85)	.036	−0.266 (−0.527, −0.006)

GAIS, Global Aesthetic Improvement Scale; HA, hyaluronic acid; PLLA, poly-L-lactic acid; SD, standard deviation.

#### Participant-Assessed GAIS

Participant-assessed GAIS scores (Table 4, Figure 4) revealed a parallel trend. At Week 4, HA was favored (2.34 ± 0.79 vs 2.04 ± 0.77; *P* = .004). No significant differences were found at Weeks 12, 24, or 36. By Week 48, however, PLLA was rated superior by participants (2.66 ± 0.85 vs 2.42 ± 0.92; *P* = .036). The trends were similar in the PPS (Supplemental Table 6).

### Safety Assessment

Mild, treatment-related AEs were reported in both groups and were generally transient and self-resolving (Table 5). The most frequent events in the PLLA group were swelling (98.4%), pain at the injection site (94.4%), erythema (93.7%), and pain at the filler site (88.9%). Rates of swelling and pain were similar between groups (all *P* > .05). However, erythema occurred more frequently in the PLLA group compared with the HA group (93.7% vs 80.8%, *P* = .002), as did bleeding at either the injection site (75.4% vs 63.2%, *P* = .041) or the filler site (62.7% vs 41.6%, *P* = .001). Conversely, palpable masses were more common in the HA group (41.6% vs 29.4%, *P* = .048). Rates of nodules and pruritus did not differ significantly between groups. All AEs were mild, resolved spontaneously without intervention, and did not require hospitalization. No treatment-related serious systemic events were observed.

**Table 5.** Adverse Events

	PLLA group (*n* = 126)		HA group (*n* = 125)		*P*–value
	No. of cases	%	No. of cases	%	
Swelling	124	98.41	118	94.40	.102
Pain (injection site)	119	94.44	113	90.40	.243
Pain (filler site)	112	88.89	114	91.20	.674
Erythema	118	93.65	101	80.80	.002
Bleeding (injection site)	95	75.40	79	63.20	.041
Bleeding (filler site)	79	62.70	52	41.60	.001
Mass	37	29.37	52	41.60	.048
Nodules	32	25.40	45	36.00	.076
Pruritus	34	26.98	29	23.20	.561

HA, hyaluronic acid; PLLA, poly-L-lactic acid.

## DISCUSSION

This multicenter, randomized, double-blind, parallel-controlled trial demonstrated that injectable PLLA microspheres provided significantly superior long-term (ie, 48 weeks) correction of moderate-to-severe NLFs compared with HA under the specific treatment protocols used in this study. Although HA achieved greater early improvement in wrinkle severity and aesthetic appearance at 4 and 12 weeks, PLLA showed a delayed onset of action but produced durable and statistically superior outcomes at 36 and 48 weeks, as confirmed by WSRS and GAIS assessments from both investigators and participants. Importantly, the safety profile of PLLA was comparable to HA, with no serious AEs reported. Together, these findings highlight distinct efficacy profiles—HA offering immediate but transient improvement under current protocols, and PLLA providing progressive and sustained correction—which may guide patient–physician decision making in clinical practice.

The temporal efficacy patterns observed in this trial are consistent with the distinct mechanism of action of HA and PLLA. As shown in Tables 2, 3 and Figures 2, 3, HA demonstrated superior short-term correction at 4 weeks, reflecting its immediate hydrophilic volumizing effect. However, this benefit diminished over time, with no significant difference at Weeks 12 and 24, and a clear decline by Week 48. In contrast, PLLA exhibited a delayed onset of action, as expected for a biostimulatory filler, but demonstrated durable and statistically superior outcomes at 36 and 48 weeks. These findings underscore a clinically relevant trade-off: HA provides immediate but transient improvement, whereas PLLA offers progressive and long-lasting correction. Importantly, this pattern should inform patient–physician discussions when selecting fillers, as patient priorities may differ between rapid visible change and extended durability of results. Although PLLA has been used as an effective dermal filler for over 2 decades, head-to-head comparative studies with HA—the most widely used dermal filler—remain limited.^12^ Previous randomized trials by Hyun et al and Yang et al demonstrated comparable volumetric and aesthetic efficacy between PLLA and HA in NLF correction and penile augmentation, respectively.^13,14^ However, these studies were limited by relatively short follow-up durations of up to 24 weeks, precluding conclusions regarding long-term outcomes. Moreover, the small sample sizes in both trials limit the generalizability and statistical robustness of their findings. In these 2 studies, temporal efficacy patterns were not observed, probably because of the limited sample size and short follow-up. A more recent multicenter randomized controlled trial by Ting et al reported superior volumetric correction with a poly-lactic acid–based filler compared with HA.^15^ However, the filler investigated differed structurally from the PLLA formulation assessed in the current study, thus precluding direct comparison. Therefore, the long-term efficacy of PLLA relative to conventional fillers, such as HA, remains inadequately characterized. The present trial addresses these knowledge gaps by evaluating the effects of PLLA up to 48 weeks postinjection, revealing a distinct temporal efficacy profile characterized by delayed onset but durable correction compared with HA. Furthermore, the study was designed with methodological rigor to enhance validity, employing central randomization, double-blinding of participants and evaluators, and outcome adjudication by an independent review committee to minimize bias. The use of both the FAS and per-protocol set (PPS) further reinforces the robustness of the efficacy findings.

Secondary endpoints further support the sustained performance of PLLA from both investigators’ and participants’ perspective. Although HA outperformed PLLA at early time points in WSRS and GAIS assessments, PLLA showed superior results at 36 and 48 weeks. This pattern, in accord with WSRS trends, underscores the biostimulatory nature of PLLA and reinforces its utility in patients prioritizing longevity of effect.

Interestingly, the frequency of treatment sessions differed significantly between groups. Most participants in the PLLA group required 3 or 4 sessions within the 12-week treatment time window, whereas the majority in the HA group achieved correction with only 1 or 2 sessions. This difference reflects the distinct mechanisms of the 2 fillers: HA provides immediate volumization with fewer sessions but shorter-lived effects, whereas PLLA depends on cumulative biostimulatory activity that necessitates more frequent early treatments yet results in more durable outcomes, which prolongs the revisiting cycle in the later treatment. Although participants in the PLLA group experienced more complementary injection sessions, cumulative injection-related discomfort and satisfaction are generally comparable to the HA group. The satisfaction is even higher in the PLLA group at 48 weeks (Supplemental Tables 7, 8). This trade-off should be considered when tailoring treatment to patient priorities. It should be noted, however, that previous work has demonstrated extended durability of HA when booster protocols are optimized. In particular, Narins et al reported persistence of NLF correction with Restylane up to 18 months when a booster injection was performed at 4.5 or 9 months.^16^ This important evidence indicates that long-term HA efficacy may be greater than suggested by single-course protocols, and direct comparison with optimized HA regimens was not within the scope of the present study. Therefore, our findings should be interpreted in this context—as characterizing the temporal efficacy profile of PLLA relative to HA under the treatment protocols evaluated—rather than as a definitive demonstration of long-term superiority.

Importantly, the safety profiles of both agents were favorable. Mild, localized AEs were reported in both groups and resolved without intervention. No systemic or serious treatment-related events were observed, reflecting the established safety of both filler types when properly administered. Notably, erythema and bleeding—either at the injection or filler site—occurred more frequently in the PLLA group, which may be attributable to the larger needle size and biostimulatory properties of PLLA under the multisession protocol.^17^ In contrast, palpable masses were reported more commonly in the HA group, possibly reflecting the immediate gel-based volumizing effect or product rheology. None of these events required clinical management. These findings reinforce that both products are safe when used appropriately, although clinicians should counsel patients regarding transient, procedure-related reactions that may be more common with PLLA during the early treatment phase.

Several limitations should be acknowledged. First, although the duration of follow-up (48 weeks) is relatively long for filler studies, extended observation beyond 1 year would be informative to determine true product longevity and potential late-onset AEs. To partially address this, we selected a follow-up period long enough to capture both the delayed onset and plateau phase of PLLA's biostimulatory effect. Second, the study design did not include a split-face comparison or patient preference assessment, which could have provided stronger within-subject and subjective comparisons; however, we mitigated this by employing central randomization, strict eligibility criteria, and matched baseline wrinkle severity across groups. Third, despite blinding safeguards, inherent product differences might have introduced partial unblinding; to minimize this risk, syringes were masked and outcome assessors were separated from the injection process. Fourth, mechanistic claims regarding stimulation are based on established literature rather than direct histologic or imaging-based evaluation in this cohort; however, the inclusion of both FAS and PPS analyses, coupled with standardized outcome measures (WSRS, GAIS), strengthens the robustness of our clinical findings.^4^ Fifth, the study population consisted exclusively of Chinese adults, which may limit generalizability to more diverse populations. Sixth, missing data occurred at several interim follow-up time points. Although all participants in the PPS completed the 48-week visit, attrition in the FAS at Weeks 4, 12, 24, and 36 may have influenced the temporal trend of treatment responses. Conservative imputation and PPS confirmation were applied to mitigate this bias, but the possibility of residual impact cannot be excluded. Finally, this study compared PLLA with HA under the treatment protocol used here, without incorporating optimization strategies for HA such as scheduled retreatment at 9 months, which has been shown to prolong efficacy up to 18 months in previous studies.^16^ Thus, our findings should be interpreted within the context of current protocols.

Future studies should extend follow-up beyond 1 year to better define the true longevity of PLLA and capture potential late-onset AEs. Inclusion of more diverse patient populations will be critical to assess generalizability across ethnicities and skin types. In addition, comparative studies against other biostimulatory agents, such as calcium hydroxyapatite, as well as combination protocols, may help clarify the relative positioning of PLLA in aesthetic practice. Importantly, direct head-to-head trials with optimized HA protocols—including scheduled retreatment—are warranted to more definitively characterize the long-term comparative efficacy of PLLA vs HA. Incorporating patient-reported outcome measures to assess satisfaction and psychosocial benefit, along with objective imaging tools such as high-frequency ultrasound or 3-dimensional volumetric imaging, may further elucidate the underlying tissue changes and improve understanding of PLLA's mechanism of action.

## CONCLUSIONS

This randomized, multicenter trial demonstrates that PLLA microspheres provide durable correction of NLFs with a safety profile comparable to HA. Under the current protocols, HA offers more immediate improvement, whereas PLLA requires multiple early treatment sessions but achieves more durable improvement up to 48 weeks. These results reflect outcomes under current HA protocols without scheduled retreatment. Therefore, PLLA and HA should be viewed as complementary treatment options, with filler selection individualized according to patient priorities for rapid vs long-term outcomes. Further studies with optimized HA retreatment schedules, extended follow-up, inclusion of diverse populations, and incorporation of patient preference measures will further refine the clinical positioning of PLLA in aesthetic practice.

## Supplemental Material

This article contains supplemental material located online at https://doi.org/10.1093/asjof/ojag001.

## Supplementary Material

ojag001_Supplementary_Data
