# Supplementary material for: Long-Term Correction of Nasolabial Folds Using Poly-L-Lactic Acid Microspheres: A Multicenter, Double-Blinded, Randomized Trial
Source: Aesthet Surg J Open Forum. 2026 Jan 13;8:ojag001. doi: 10.1093/asjof/ojag001 (PMC12903950; doi:10.1093/asjof/ojag001)
Supplement: ojag001_Supplementary_Data [file ojag001_supplementary_data.zip › Appendix.docx]

**Long-Term Correction of Nasolabial Folds Using Poly-L-Lactic Acid Microspheres: A Multicenter, Double-Blind, Randomized Trial-Supplemental Materials**

1. **Participant inclusion and exclusion criteria**

**Inclusion Criteria:**

1. Age ≥ 18 years, both male and female;
2. A score of 3–4 on the WSRS for NLFs;
3. Willingness to refrain from using other aesthetic procedures during the study;
4. Ability to understand and comply with study requirements, including completing all follow-up visits;
5. Voluntary participation and signed informed consent.

**Exclusion Criteria:**

1. Pregnant or breastfeeding women, or those planning to become pregnant during the study;
2. Women of childbearing potential who do not agree to use medically approved contraceptive measures during the trial;
3. Participants with visual impairments that could affect assessment;
4. Clinically significant abnormalities in laboratory tests that render participants unsuitable for facial filler treatment;
5. Positive results for hepatitis B surface antigen (HBsAg), hepatitis C antibody (anti-HCV), HIV antibody (anti-HIV), or syphilis-specific antibody (anti-TP);
6. Scars or skin diseases in the treatment area, or active inflammation and/or unhealed wounds;
7. Prior permanent dermal filler treatments in the NLF;
8. Previous facial lifting surgeries or thread lifting procedures within the year preceding the study;
9. Treatments with hyaluronic acid or other semi-permanent fillers in the NLF within the past year;
10. Other wrinkle treatments (e.g., botulinum toxin, laser treatments) within the six months before screening;
11. Use of anticoagulants or antiplatelet agents within 14 days prior to treatment;
12. History of severe allergies, or known allergies to study products or lidocaine;
13. Significant organ disease or autoimmune disorders;
14. History of keloid formation;
15. Participation in other clinical trials within 30 days prior to screening;
16. Other conditions deemed unsuitable by the investigator.

Patients who met the above criteria were included in this study at their first visit.

1. **Sample size determination**

This was a randomized controlled study to evaluate the efficacy of PLLA in treating nasolabial folds. The sample size was determined by the following formula:

$$n_{T}=n_{C}=\frac{\left( Z_{1-\alpha/2}+Z_{1-\beta} \right)^{2}\left[ P_{C}\left( 1-P_{C} \right)+P_{T}\left( 1-P_{T} \right) \right]}{\left( \left| D \right|-\Delta\right)^{2}}$$

in which n*_T_* was the sample size of the treatment group; n*_c_* was the sample size of the control group; *P_T_* was the efficacy of the treatment group; *Pc* was the efficacy of the control group; |D|=| *P_T_* - *Pc* |; and Δ was the noninferiority margin.

According to the purpose of this clinical trial and the results of previous clinical trials of control products, the specific parameter settings for sample content estimation are as follows:

(1) The one-sided test level of superiority α=0.015;

(2) The test confidence is 80%, then β=0.20;

(3) The expected effective rate of the experimental group is 20% lower than that of the control group, then D=20%;

(4) The expected effective rate of the control group is 55%, then *P_T_* =0.75, *Pc* =0.55.

After calculation, it is found that: n*_T_* = n*_c_*=99, considering the dropout rate of 20%, the sample size of each group is 124 cases.

The sample size was finally determined: according to the principle of taking the largest sample size, the sample size of each group was selected as 124 cases, and the total number of samples was 248 cases, including 124 cases in the experimental group and 124 cases in the control group.
