# Supplementary material for: Long-Term Correction of Nasolabial Folds Using Poly-L-Lactic Acid Microspheres: A Multicenter, Double-Blinded, Randomized Trial
Source: Aesthet Surg J Open Forum. 2026 Jan 13;8:ojag001. doi: 10.1093/asjof/ojag001 (PMC12903950; doi:10.1093/asjof/ojag001)
Supplement: ojag001_Supplementary_Data [file ojag001_supplementary_data.zip › Supplemental Table 1.docx]

**Supplemental Table 1. Wrinkle severity rating scale**

| **Grade** | **Category** | **Description of characteristics** |
| --- | --- | --- |
| 1 | Absent | No visible nasolabial fold; continuous skin line. |
| 2 | Mild | Shallow but visible nasolabial fold with a slight indentation; minor facial feature; |
| 3 | Moderate | Moderately deep nasolabial fold; clear facial feature visible at normal appearance but not when stretched; |
| 4 | Severe | Very long and deep nasolabial fold; prominent facial feature; <2 mm visible fold when stretched; |
| 5 | Extreme | Extremely deep and long nasolabial fold, detrimental to facial appearance; 2–4mm visible V-shaped fold when stretched; |
