# Supplementary material for: Long-Term Correction of Nasolabial Folds Using Poly-L-Lactic Acid Microspheres: A Multicenter, Double-Blinded, Randomized Trial
Source: Aesthet Surg J Open Forum. 2026 Jan 13;8:ojag001. doi: 10.1093/asjof/ojag001 (PMC12903950; doi:10.1093/asjof/ojag001)
Supplement: ojag001_Supplementary_Data [file ojag001_supplementary_data.zip › Supplemental Table 2.docx]

**Supplemental Table 2. Frequency of treatment between groups (FAS)**

| **Frequency** | **PLLA** | **HA** | **Statistic value (Rank-sum tests)** | ***P* value** |
| --- | --- | --- | --- | --- |
| 1 , n(%) | 12(10.08) | 27(22.88) | 9800.0 | **<.0001** |
| 2, n(%) | 18(15.13) | 67(56.78) |  |  |
| 3, n(%) | 26(21.85) | 20(16.95) |  |  |
| 4, n(%) | 63(52.94) | 4(3.39) |  |  |
| Net (Missing) | 119(0) | 118(0) |  |  |
