# Supplementary material for: Long-Term Correction of Nasolabial Folds Using Poly-L-Lactic Acid Microspheres: A Multicenter, Double-Blinded, Randomized Trial
Source: Aesthet Surg J Open Forum. 2026 Jan 13;8:ojag001. doi: 10.1093/asjof/ojag001 (PMC12903950; doi:10.1093/asjof/ojag001)
Supplement: ojag001_Supplementary_Data [file ojag001_supplementary_data.zip › Supplemental Table 3.docx]

**Supplemental Table 3. Frequency of treatment between groups (PPS)**

| **Frequency** | **PLLA** | **HA** | **Statistic value (Rank-sum tests)** | ***P* value** |
| --- | --- | --- | --- | --- |
| 1, n(%) | 7(6.31) | 27(23.08) | 17048 | **<.0001** |
| 2, n(%) | 17(15.32) | 66(56.41) |  |  |
| 3, n(%) | 24(21.62) | 20(17.09) |  |  |
| 4, n(%) | 63(56.76) | 4(3.42) |  |  |
| Net (Missing) | 111(0) | 117(0) |  |  |
