# Supplementary material for: Long-Term Correction of Nasolabial Folds Using Poly-L-Lactic Acid Microspheres: A Multicenter, Double-Blinded, Randomized Trial
Source: Aesthet Surg J Open Forum. 2026 Jan 13;8:ojag001. doi: 10.1093/asjof/ojag001 (PMC12903950; doi:10.1093/asjof/ojag001)
Supplement: ojag001_Supplementary_Data [file ojag001_supplementary_data.zip › Supplemental Table 4.docx]

**Supplemental Table 4. Comparison of WSRS effective ratio between PLLA and HA groups (PPS).**

| **Group** | **N** | **Effective ratio (%)** | **Difference (95%CI)** | ***P* value** |
| --- | --- | --- | --- | --- |
| **Week 4** |  |  |  |  |
| PLLA (N=102) | 94 | 92.16 | -6.92% (-13.83%, -1.29%) | **0.0162** |
| HA (N=108) | 107 | 99.07 |  |  |
| **Week 12** |  |  |  |  |
| PLLA (N=105) | 102 | 97.14 | -0.95% (-6.34%, 4.18%) | 1.000 |
| HA (N=105) | 103 | 98.10 |  |  |
| **Week 24** |  |  |  |  |
| PLLA (N=109) | 105 | 96.33 | 3.47% (-2.94%, 10.18%) | 0.2546 |
| HA (N=112) | 104 | 92.86 |  |  |
| **Week 36** |  |  |  |  |
| PLLA (N=108) | 106 | 98.15 | 18.84% (10.95%, 27.20%) | **<.0001** |
| HA (N=116) | 92 | 79.31 |  |  |
| **Week 48** |  |  |  |  |
| PLLA (N=111) | 106 | 95.50 | 35.67% (25.4%, 45.08%) | **<.0001** |
| HA (N=117) | 70 | 59.83 |  |  |

WSRS scores were assessed by blinded independent evaluators; PPS, Per-Protocol Set; CI, confidence interval.
