# Supplementary material for: Long-Term Correction of Nasolabial Folds Using Poly-L-Lactic Acid Microspheres: A Multicenter, Double-Blinded, Randomized Trial
Source: Aesthet Surg J Open Forum. 2026 Jan 13;8:ojag001. doi: 10.1093/asjof/ojag001 (PMC12903950; doi:10.1093/asjof/ojag001)
Supplement: ojag001_Supplementary_Data [file ojag001_supplementary_data.zip › Supplemental Table 5.docx]

**Supplemental Table 5. Comparison of WSRS improvement from baseline of PLLA and HA groups (PPS).**

| **WSRS** | **PLLA** | |  | **HA** | |  | ***P* value** | **Standardized effect size** |  |
| --- | --- | --- | --- | --- | --- | --- | --- | --- | --- |
|  | **N** | **Value of change，Mean(SD)** |  | **N** | **Value of change，Mean(SD)** |  |  |  |  |
| Week 4 | 102 | -1.33 (0.63) |  | 108 | -1.75 (0.60) |  | **<.0001** | 0.676(0.397,0.956) |  |
| Week 12 | 105 | -1.43 (0.62) |  | 105 | -1.56 (0.65) |  | 0.136 | 0.210(-0.062,0.483) |  |
| Week 24 | 109 | -1.32 (0.56) |  | 112 | -1.38 (0.65) |  | 0.409 | 0.089(-0.176,0.354) |  |
| Week 36 | 108 | -1.31 (0.54) |  | 116 | -1.11 (0.77) |  | **0.025** | -0.306(-0.568,-0.044) |  |
| Week 48 | 111 | -1.32 (0.61) |  | 117 | -0.84 (0.84) |  | **<.0001** | -0.665(-0.925,-0.404) |  |

WSRS scores were assessed by blinded independent evaluators; PPS, Per-Protocol Set; SD, standard deviation.
