# Supplementary material for: Long-Term Correction of Nasolabial Folds Using Poly-L-Lactic Acid Microspheres: A Multicenter, Double-Blinded, Randomized Trial
Source: Aesthet Surg J Open Forum. 2026 Jan 13;8:ojag001. doi: 10.1093/asjof/ojag001 (PMC12903950; doi:10.1093/asjof/ojag001)
Supplement: ojag001_Supplementary_Data [file ojag001_supplementary_data.zip › Supplemental Table 6.docx]

**Supplemental Table 6. Comparison of GAIS between PLLA and HA groups (PPS).**

| **GAIS** | **PLLA** | |  | **HA** | |  | ***P* value** | **Standardized effect size** |  |
| --- | --- | --- | --- | --- | --- | --- | --- | --- | --- |
|  | **N** | **Score，Mean(SD)** |  | **N** | **Score，Mean(SD)** |  |  |  |  |
| **Investigator-assessed** | | | | | | | | |  |
| Week 4 | 105 | 2.25 (0.76) |  | 107 | 1.84 (0.65) |  | **<.0001** | 0.578(0.302,0.854) |  |
| Week 12 | 107 | 2.23 (0.80) |  | 108 | 2.00 (0.67) |  | **0.027** | 0.318(0.047,0.588) |  |
| Week 24 | 110 | 2.27 (0.73) |  | 113 | 2.26 (0.70) |  | 0.961 | 0.022(-0.241,0.286) |  |
| Week 36 | 108 | 2.24 (0.71) |  | 116 | 2.48 (0.69) |  | **0.011** | -0.346(-0.611,-0.081) |  |
| Week 48 | 111 | 2.42 (0.67) |  | 117 | 2.77 (0.79) |  | **0.001** | -0.471(-0.735,-0.207) |  |
| **Participant-assessed** | | | | | | | | |  |
| Week 4 | 105 | 2.31 (0.79) |  | 107 | 2.05 (0.77) |  | **0.013** | 0.344(0.071,0.616) |  |
| Week 12 | 107 | 2.37 (0.84) |  | 108 | 2.31 (0.68) |  | 0.397 | 0.089(-0.180,0.359) |  |
| Week 24 | 110 | 2.39 (0.85) |  | 113 | 2.35 (0.82) |  | 0.742 | 0.055(-0.209,0.319) |  |
| Week 36 | 108 | 2.39 (0.88) |  | 116 | 2.45 (0.82) |  | 0.656 | -0.070(-0.333,0.193) |  |
| Week 48 | 111 | 2.42 (0.93) |  | 117 | 2.66 (0.85) |  | **0.040** | -0.263(-0.525,-0.002) |  |

WSRS scores were assessed by blinded independent evaluators; PPS, Per-Protocol Set; SD, standard deviation.
