# Supplementary material for: Long-Term Correction of Nasolabial Folds Using Poly-L-Lactic Acid Microspheres: A Multicenter, Double-Blinded, Randomized Trial
Source: Aesthet Surg J Open Forum. 2026 Jan 13;8:ojag001. doi: 10.1093/asjof/ojag001 (PMC12903950; doi:10.1093/asjof/ojag001)
Supplement: ojag001_Supplementary_Data [file ojag001_supplementary_data.zip › Supplemental Table 7.docx]

**Supplemental Table 7. Participant satisfaction (FAS)**

| **Satisfaction scale** | **PLLA** | **HA** | **Statistic value (Rank-sum tests)** | ***P* value** |
| --- | --- | --- | --- | --- |
| **Immediately after 1st injection** |  |  | 14458 | 0.4064 |
| 0.unsatisfied, n(%) | 2(1.68) | 0(0) |  |  |
| 1.satisfied, n(%) | 22(18.49) | 16(13.56) |  |  |
| 2.good, n(%) | 17(14.29) | 20(16.95) |  |  |
| 3.very good, n(%) | 29(24.37) | 30(25.42) |  |  |
| 4.extremely good, n(%) | 49(41.18) | 52(44.07) |  |  |
| Net (Missing) | 119(0) | 118(0) |  |  |
| **Immediately after 2nd injection** |  |  | 9280.0 | 0.5597 |
| 0.unsatisfied, n(%) | 1(0.93) | 0(0) |  |  |
| 1.satisfied, n(%) | 20(18.69) | 18(19.78) |  |  |
| 2.good, n(%) | 22(20.56) | 9(9.89) |  |  |
| 3.very good, n(%) | 28(26.17) | 34(37.36) |  |  |
| 4.extremely good, n(%) | 36(33.64) | 30(32.97) |  |  |
| Net (Missing) | 107(12) | 91(27) |  |  |
| **Immediately after 3rd injection** |  |  | 1327.5 | 0.7699 |
| 0.unsatisfied, n(%) | 1(1.12) | 0(0) |  |  |
| 1.satisfied, n(%) | 17(19.10) | 3(12.50) |  |  |
| 2.good, n(%) | 16(17.98) | 5(20.83) |  |  |
| 3.very good, n(%) | 20(22.47) | 10(41.67) |  |  |
| 4.extremely good, n(%) | 35(39.33) | 6(25.00) |  |  |
| Net (Missing) | 89(30) | 24(94) |  |  |
| **Immediately after 4th injection** |  |  | 75.50 | 0.0884 |
| 0.unsatisfied, n(%) | 0(0) | 0(0) |  |  |
| 1.satisfied, n(%) | 12(18.75) | 1(25.00) |  |  |
| 2.good, n(%) | 8(12.50) | 2(50.00) |  |  |
| 3.very good, n(%) | 15(23.44) | 1(25.00) |  |  |
| 4.extremely good, n(%) | 29(45.31) | 0(0) |  |  |
| Net (Missing) | 64(55) | 4(114) |  |  |
| **Week 4** |  |  | 11849 | 0.9464 |
| 0.unsatisfied, n(%) | 3(2.70) | 2(1.85) |  |  |
| 1.satisfied, n(%) | 19(17.12) | 21(19.44) |  |  |
| 2.good, n(%) | 30(27.03) | 30(27.78) |  |  |
| 3.very good, n(%) | 36(32.43) | 30(27.78) |  |  |
| 4.extremely good, n(%) | 23(20.72) | 25(23.15) |  |  |
| Net (Missing) | 111(8) | 108(10) |  |  |
| **Week 12** |  |  | 12060 | 0.9745 |
| 0.unsatisfied, n(%) | 3(2.70) | 2(1.83) |  |  |
| 1.satisfied, n(%) | 19(17.12) | 17(15.60) |  |  |
| 2.good, n(%) | 27(24.32) | 32(29.36) |  |  |
| 3.very good, n(%) | 41(36.94) | 36(33.03) |  |  |
| 4.extremely good, n(%) | 21(18.92) | 22(20.18) |  |  |
| Net (Missing) | 111(8) | 109(9) |  |  |
| **Week 24** |  |  | 13190 | 0.5199 |
| 0.unsatisfied, n(%) | 4(3.54) | 5(4.39) |  |  |
| 1.satisfied, n(%) | 19(16.81) | 17(14.91) |  |  |
| 2.good, n(%) | 28(24.78) | 37(32.46) |  |  |
| 3.very good, n(%) | 40(35.40) | 35(30.70) |  |  |
| 4.extremely good, n(%) | 22(19.47) | 20(17.54) |  |  |
| Net (Missing) | 113(6) | 114(4) |  |  |
| **Week 36** |  |  | 13283 | 0.0556 |
| 0.unsatisfied, n(%) | 5(4.59) | 7(5.98) |  |  |
| 1.satisfied, n(%) | 18(16.51) | 21(17.95) |  |  |
| 2.good, n(%) | 28(25.69) | 40(34.19) |  |  |
| 3.very good, n(%) | 29(26.61) | 33(28.21) |  |  |
| 4.extremely good, n(%) | 29(26.61) | 16(13.68) |  |  |
| Net (Missing) | 109(10) | 117(1) |  |  |
| **Week 48** |  |  | 14039 | **0.0429** |
| 0.unsatisfied, n(%) | 6(5.31) | 6(5.13) |  |  |
| 1.satisfied, n(%) | 17(15.04) | 21(17.95) |  |  |
| 2.good, n(%) | 27(23.89) | 39(33.33) |  |  |
| 3.very good, n(%) | 36(31.86) | 38(32.48) |  |  |
| 4.extremely good, n(%) | 27(23.89) | 13(11.11) |  |  |
| Net (Missing) | 113(6) | 117(1) |  |  |
