# Supplementary material for: Long-Term Correction of Nasolabial Folds Using Poly-L-Lactic Acid Microspheres: A Multicenter, Double-Blinded, Randomized Trial
Source: Aesthet Surg J Open Forum. 2026 Jan 13;8:ojag001. doi: 10.1093/asjof/ojag001 (PMC12903950; doi:10.1093/asjof/ojag001)
Supplement: ojag001_Supplementary_Data [file ojag001_supplementary_data.zip › Supplemental Table 8.docx]

**Supplemental Table 8. Participant satisfaction (PPS)**

| **Satisfaction scale** | **PLLA** | **HA** | **Statistic**  **(Rank-sum tests)** | ***P* value** |
| --- | --- | --- | --- | --- |
| **Immediately after 1st injection** |  |  | 12338 | 0.4309 |
| 0.unsatisfied, n(%) | 2(1.80) | 0(0) |  |  |
| 1.satisfied, n(%) | 19(17.12) | 16(13.68) |  |  |
| 2.good, n(%) | 17(15.32) | 19(16.24) |  |  |
| 3.very good, n(%) | 27(24.32) | 30(25.64) |  |  |
| 4.extremely good, n(%) | 46(41.44) | 52(44.44) |  |  |
| Net (Missing) | 111(0) | 117(0) |  |  |
| **Immediately after 2nd injection** |  |  | 8923.5 | 0.6925 |
| 0.unsatisfied, n(%) | 1(0.96) | 0(0) |  |  |
| 1.satisfied, n(%) | 18(17.31) | 18(20.00) |  |  |
| 2.good, n(%) | 22(21.15) | 9(10.00) |  |  |
| 3.very good, n(%) | 27(25.96) | 33(36.67) |  |  |
| 4.extremely good, n(%) | 36(34.62) | 30(33.33) |  |  |
| Net (Missing) | 104(7) | 90(27) |  |  |
| **Immediately after 3rd injection** |  |  | 1294.0 | 0.7116 |
| 0.unsatisfied, n(%) | 1(1.15) | 0(0) |  |  |
| 1.satisfied, n(%) | 16(18.39) | 3(12.50) |  |  |
| 2.good, n(%) | 16(18.39) | 5(20.83) |  |  |
| 3.very good, n(%) | 19(21.84) | 10(41.67) |  |  |
| 4.extremely good, n(%) | 35(40.23) | 6(25.00) |  |  |
| Net (Missing) | 87(24) | 24(93) |  |  |
| **Immediately after 4th injection** |  |  | 75.50 | 0.0884 |
| 0.unsatisfied, n(%) | 0(0) | 0(0) |  |  |
| 1.satisfied, n(%) | 12(18.75) | 1(25.00) |  |  |
| 2.good, n(%) | 8(12.50) | 2(50.00) |  |  |
| 3.very good, n(%) | 15(23.44) | 1(25.00) |  |  |
| 4.extremely good, n(%) | 29(45.31) | 0(0) |  |  |
| Net (Missing) | 64(47) | 4(113) |  |  |
| **Week 4** |  |  | 11225 | 0.9225 |
| 0.unsatisfied, n(%) | 3(2.86) | 2(1.87) |  |  |
| 1.satisfied, n(%) | 17(16.19) | 21(19.63) |  |  |
| 2.good, n(%) | 29(27.62) | 29(27.10) |  |  |
| 3.very good, n(%) | 34(32.38) | 30(28.04) |  |  |
| 4.extremely good, n(%) | 22(20.95) | 25(23.36) |  |  |
| Net (Missing) | 105(6) | 107(10) |  |  |
| **Week 12** |  |  | 11614 | 0.8966 |
| 0.unsatisfied, n(%) | 3(2.80) | 2(1.85) |  |  |
| 1.satisfied, n(%) | 17(15.89) | 17(15.74) |  |  |
| 2.good, n(%) | 26(24.30) | 31(28.70) |  |  |
| 3.very good, n(%) | 40(37.38) | 36(33.33) |  |  |
| 4.extremely good, n(%) | 21(19.63) | 22(20.37) |  |  |
| Net (Missing) | 107(4) | 108(9) |  |  |
| **Week 24** |  |  | 12615 | 0.5259 |
| 0.unsatisfied, n(%) | 4(3.64) | 4(3.54) |  |  |
| 1.satisfied, n(%) | 18(16.36) | 17(15.04) |  |  |
| 2.good, n(%) | 27(24.55) | 37(32.74) |  |  |
| 3.very good, n(%) | 39(35.45) | 35(30.97) |  |  |
| 4.extremely good, n(%) | 22(20.00) | 20(17.70) |  |  |
| Net (Missing) | 110(1) | 113(4) |  |  |
| **Week 36** |  |  | 13055 | 0.0543 |
| 0.unsatisfied, n(%) | 5(4.63) | 7(6.03) |  |  |
| 1.satisfied, n(%) | 18(16.67) | 21(18.10) |  |  |
| 2.good, n(%) | 27(25.00) | 39(33.62) |  |  |
| 3.very good, n(%) | 29(26.85) | 33(28.45) |  |  |
| 4.extremely good, n(%) | 29(26.85) | 16(13.79) |  |  |
| Net (Missing) | 108(3) | 116(1) |  |  |
| **Week 48** |  |  | 13569 | 0.0558 |
| 0.unsatisfied, n(%) | 6(5.41) | 6(5.17) |  |  |
| 1.satisfied, n(%) | 17(15.32) | 21(18.10) |  |  |
| 2.good, n(%) | 27(24.32) | 38(32.76) |  |  |
| 3.very good, n(%) | 34(30.63) | 38(32.76) |  |  |
| 4.extremely good, n(%) | 27(24.32) | 13(11.21) |  |  |
| Net (Missing) | 111(0) | 116(1) |  |  |
