# Supplementary figures and images for: Long-Term Correction of Nasolabial Folds Using Poly-L-Lactic Acid Microspheres: A Multicenter, Double-Blinded, Randomized Trial
Source: Aesthet Surg J Open Forum. 2026 Jan 13;8:ojag001. doi: 10.1093/asjof/ojag001 (PMC12903950; doi:10.1093/asjof/ojag001)

**Supplemental Figure 1. The WSRS improvement from baseline of PLLA and HA groups.**P* <0.05, ***P* <0.01, ****P* <0.001, *****P* <0.0001.**


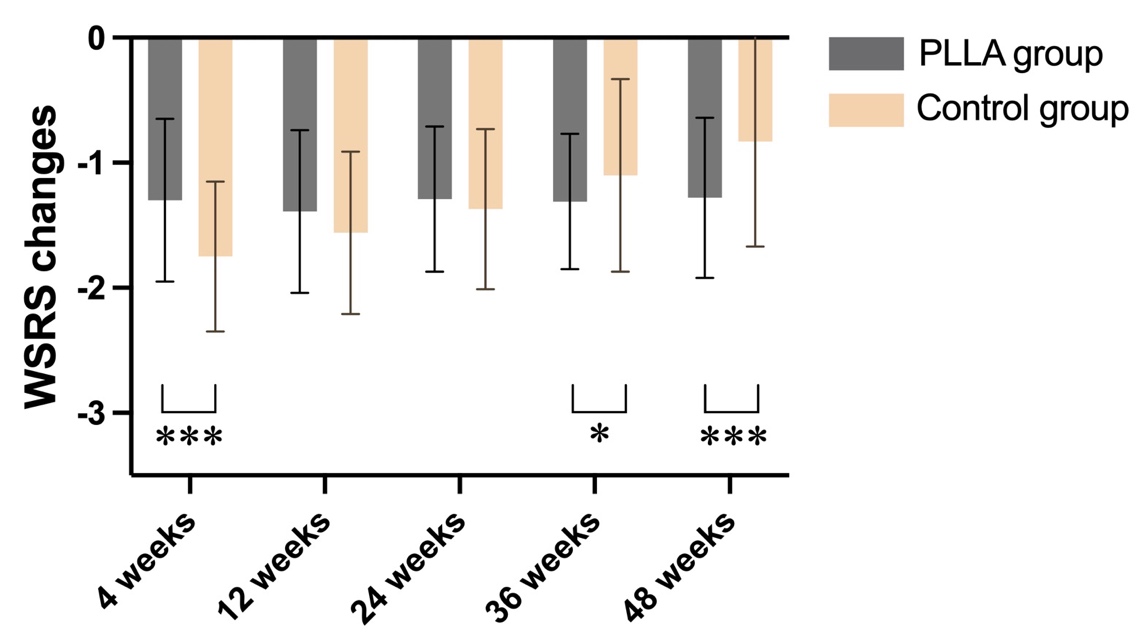

Supplement: ojag001_Supplementary_Data [file ojag001_supplementary_data.zip › Supplemental Figure 1.docx]
